# Supplementary material for: Impact of monitor unit optimization in volumetric modulated arc therapy planning for nasopharyngeal carcinoma
Source: PLoS One. 2025 Jun 25;20(6):e0327153. doi: 10.1371/journal.pone.0327153 (PMC12193012; doi:10.1371/journal.pone.0327153)
Supplement: Attachment — Submitted filename: Reviewers comments.pdf [file pone.0327153.s002.pdf]

Manuscript: PONE-D-25-12726

Research on the Impact of Monitor Unit Optimization in Volumetric Modulated Arc Therapy Planning for Nasopharyngeal Carcinoma

Comments:

I appreciate the work done in this article. An optimized MU can be a good choice to balance TV coverage and OAR doses. My advice to the author is as follows.

1. In the manuscript draft section, the Author mentioned that the patient's data used in the study was treated between October 23 to March 24. In contrast, the main abstract, manuscript, and ethics statement were mentioned as October 2020 to March 2021.
2. In the main manuscript, under the heading 'VMAT planning,' how did the author decide to take the maximum MU 30 %?, Can it be more elaborate?
3. Oar is written in small letters. It must be in capital letters.
4. Under the heading "Comparison of the target and OAR doses…." The sentence "excluding setup time and gantry rotation time…." Can be written as excluding the setup time and **gantry repositioning time, or beam mode up time**.
5. Under the heading " pretreatment plan verification," **10% threshold** in the 3% 3mm criteria? I doubt it is given in the cited reference 24.
6. Table 4 can be rearranged.
7. In Tables 5 and 6, what are t1, t2, and t3? The author must mention it and elaborate on these terms.
