# Supplementary material for: Impact of monitor unit optimization in volumetric modulated arc therapy planning for nasopharyngeal carcinoma
Source: PLoS One. 2025 Jun 25;20(6):e0327153. doi: 10.1371/journal.pone.0327153 (PMC12193012; doi:10.1371/journal.pone.0327153)
Supplement: Attachment — Submitted filename: PONE-D-25-12726_reviewer.pdf [file pone.0327153.s003.pdf]

PLOS ONE

Research on the Impact of Monitor Unit Optimization in Volumetric Modulated Arc Therapy Planning for Nasopharyngeal Carcinoma

--Manuscript Draft--

**Manuscript Number:**	PONE-D-25-12726
**Article Type:**	Research Article
**Full Title:**	Research on the Impact of Monitor Unit Optimization in Volumetric Modulated Arc Therapy Planning for Nasopharyngeal Carcinoma
**Short Title:**	Research on the Impact of Monitor Unit Optimization in VMAT Planning for NPC
**Corresponding Author:**	huaqu zeng, M.D.<br>Gaozhou People's Hospital<br>Gaozhou, CHINA
**Keywords:**	Nasopharyngeal Carcinoma; volumetric modulated arc therapy; monitor units optimization; dosimetry
**Abstract:**	Purpose<br><br>To investigate the impact of monitor units (MUs) optimization on volumetric modulated arc therapy (VMAT) plan for nasopharyngeal carcinoma (NPC).<br><br>Methods<br><br>A retrospective analysis was conducted on 21 NPC patients treated between October 2023 and March 2024. Dual-arc VMAT plan were designed for each patient using photon optimization algorithms without the monitor units objective (MUO) tool, labeled as the base plan. The clinical base plan was then re-optimized using the MUO tool with the Maximum MU parameter set to 30% of the base plans' total MUs and Strength parameters set to 50, 80, and 100, resulting in plan S50, S80, and S100. Comparisons were made between these plans and the base plan in terms of target and organ-at-risk (OAR) dose distributions, MUs, beam delivery time, and gamma passing rates. Statistical analysis was performed using SPSS 17.0.<br><br>Results<br><br>In plan S100, the D98% for target PCTV2 decreased by more than 4% in four patients compared to the base plan. In plan S80, the Dmax for target PGTV and PGTVnd and D98% for target PCTV2 decreased by more than 3% but less than 4% in two patients, while other target dose parameters changed by less than 2%. Compared to the base plan, plan S50, S80, and S100 increased the Dmax for the brainstem, with statistically significant differences ($P < 0.05$), but the maximum increase did not exceed 1.5%. Plan S50 reduced the D50% and Dmean for both parotid glands, while plan S80 reduced the Dmean for both parotids and the D50% for the left parotid, with statistically significant differences ($P < 0.001$). Plan S100 increased the D50% and Dmean for both parotids and the Dmax for the spinal cord, with statistically significant differences ($P < 0.05$). Plan S80 and S100 increased the V40 for the thyroid, with statistically significant differences ($P < 0.05$). Compared to the base plan, plan S50, S80, and S100 reduced MUs by an average of 5.1%, 21.4%, and 30.9%, respectively, while the average beam delivery time remained around 2.5 minutes for four plans. Under the 1%/1 mm condition, the gamma passing rate for base plan, S50, S80, and S100 increased sequentially.<br><br>Conclusion<br><br>Applying MUs optimization in VMAT plan for NPC can effectively reduce MUs while maintaining target dose coverage and OAR protection, improving gamma passing rate under 1%/1 mm conditions.
**Order of Authors:**	huaqu zeng, M.D.
	Zhen Li

Powered by Editorial Manager® and ProduXion Manager® from Aries Systems Corporation

	Zongyou Chen
	Shukui Tang
	Qifu Lin
	Minzhi Zhong
	Zunbei Wen
**Additional Information:**	
**Question**	**Response**
**Financial Disclosure**<br><br>Enter a financial disclosure statement that describes the sources of funding for the work included in this submission. Review the submission guidelines for detailed requirements. View published research articles from *PLOS ONE* for specific examples.<br><br>This statement is required for submission and **will appear in the published article** if the submission is accepted. Please make sure it is accurate.<br><br>**Funded studies**<br>Enter a statement with the following details:<br>• Initials of the authors who received each award<br>• Grant numbers awarded to each author<br>• The full name of each funder<br>• URL of each funder website<br>• Did the sponsors or funders play any role in the study design, data collection and analysis, decision to publish, or preparation of the manuscript?<br><br>Did you receive funding for this work?	The author(s) received no specific funding for this work.
**Competing Interests**<br><br>Use the instructions below to enter a competing interest statement for this submission. On behalf of all authors, disclose any competing interests that could be perceived to bias this work—acknowledging all financial support and any other relevant financial or non-financial competing interests.	The authors have declared that no competing interests exist.

This statement is required for submission and **will appear in the published article** if the submission is accepted. Please make sure it is accurate and that any funding sources listed in your Funding Information later in the submission form are also declared in your Financial Disclosure statement.<br><br>View published research articles from *PLOS ONE* for specific examples.<br><br>**NO authors have competing interests**<br><br>Enter: *The authors have declared that no competing interests exist.*<br><br>**Authors with competing interests**<br><br>Enter competing interest details beginning with this statement:<br><br>*I have read the journal's policy and the authors of this manuscript have the following competing interests: [insert competing interests here]*<br><br>* typeset	
**Ethics Statement**<br><br>Enter an ethics statement for this submission. This statement is required if the study involved:<br><br>• Human participants<br>• Human specimens or tissue<br>• Vertebrate animals or cephalopods<br>• Vertebrate embryos or tissues<br>• Field research<br><br>Write "N/A" if the submission does not require an ethics statement.<br><br>General guidance is provided below. Consult the submission guidelines for detailed instructions. **Make sure that all information entered here is included in the**	After receiving approval from the institutional review board at Gaozhou People's Hospital(GYLLYJ-2021043) , a total of 21 patients with NPC treated using the VMAT technique from October 1, 2020 to March 1, 2021 were retrospectively enrolled for this study. All patient data used in the study were anonymized, and informed consent was waived since the plans in this study were research use only.

**Methods section of the manuscript.**<br><br>**Format for specific study types**<br><br>**Human Subject Research (involving human participants and/or tissue)**<br>• Give the name of the institutional review board or ethics committee that approved the study<br>• Include the approval number and/or a statement indicating approval of this research<br>• Indicate the form of consent obtained (written/oral) or the reason that consent was not obtained (e.g. the data were analyzed anonymously)<br><br>**Animal Research (involving vertebrate animals, embryos or tissues)**<br>• Provide the name of the Institutional Animal Care and Use Committee (IACUC) or other relevant ethics board that reviewed the study protocol, and indicate whether they approved this research or granted a formal waiver of ethical approval<br>• Include an approval number if one was obtained<br>• If the study involved *non-human primates*, add *additional details* about animal welfare and steps taken to ameliorate suffering<br>• If anesthesia, euthanasia, or any kind of animal sacrifice is part of the study, include briefly which substances and/or methods were applied<br><br>**Field Research**<br><br>Include the following details if this study involves the collection of plant, animal, or other materials from a natural setting:<br>• Field permit number<br>• Name of the institution or relevant body that granted permission	
**Data Availability**<br><br>Authors are required to make all data underlying the findings described fully available, without restriction, and from the time of publication. PLOS allows rare exceptions to address legal and ethical concerns. See the PLOS Data Policy and FAQ for detailed information.	Yes - all data are fully available without restriction

Powered by Editorial Manager® and ProduXion Manager® from Aries Systems Corporation

A Data Availability Statement describing where the data can be found is required at submission. Your answers to this question constitute the Data Availability Statement and **will be published in the article**, if accepted.<br><br>**Important:** Stating 'data available on request from the author' is not sufficient. If your data are only available upon request, select 'No' for the first question and explain your exceptional situation in the text box.<br><br>Do the authors confirm that all data underlying the findings described in their manuscript are fully available without restriction?	
**Describe where the data may be found in full sentences. If you are copying our sample text, replace any instances of XXX with the appropriate details.**<br><br>• If the data are **held or will be held in a public repository**, include URLs, accession numbers or DOIs. If this information will only be available after acceptance, indicate this by ticking the box below. For example: *All XXX files are available from the XXX database (accession number(s) XXX, XXX.*).<br>• If the data are all contained **within the manuscript and/or Supporting Information files**, enter the following: *All relevant data are within the manuscript and its Supporting Information files.*<br>• If neither of these applies but you are able to provide **details of access elsewhere**, with or without limitations, please do so. For example:<br><br>*Data cannot be shared publicly because of [XXX]. Data are available from the XXX Institutional Data Access / Ethics Committee (contact via XXX) for researchers who meet the criteria for access to confidential data.*<br><br>*The data underlying the results*	All relevant data are within the manuscript and its Supporting Information files.

presented in the study are available from (include the name of the third party and contact information or URL).<br>• This text is appropriate if the data are owned by a third party and authors do not have permission to share the data.<br><br>* typeset	
Additional data availability information:	Tick here if the URLs/accession numbers/DOIs will be available only after acceptance of the manuscript for publication so that we can ensure their inclusion before publication.

Manuscript Click here to access/download;Manuscript;Manuscript.docx

Research on the Impact of Monitor Unit Optimization in Volumetric Modulated Arc Therapy Planning for Nasopharyngeal Carcinoma

Huaqu Zeng[1*], Zhen Li[2], Zongyou Chen[1], Shukui Tang[1], Qifu Lin[1], Minzhi Zhong[3], Zunbei Wen[1],

1 Radiotherapy Center, Gaozhou People's Hospital, Gaozhou, Guangdong, China,

2 Department of Oncology Radiotherapy, Zhongshan City People's Hospital, Zhongshan, Guangdong, China

3 Department of Radiology, Guangzhou Red Cross Hospital, Guangzhou, Guangdong, China

***756660752@qq.com**

Abstract

Purpose

To investigate the impact of monitor units (MUs) optimization on volumetric modulated arc therapy (VMAT) plan for nasopharyngeal carcinoma (NPC).

Methods

A retrospective analysis was conducted on 21 NPC patients treated between October 2020 and March 2021 Dual-arc VMAT plan were designed for each patient using photon optimization algorithms without the monitor units objective (MUO) tool, labeled as the base plan. The clinical base plan was then re-optimized using the MUO tool with the Maximum MU parameter set to 30% of the base plans' total MUs and Strength parameters set to 50, 80, and 100, resulting in plan S_{50}, S_{80}, and S_{100}. Comparisons were made between these plans and the base plan in terms of target and organ-at-risk (OAR) dose distributions, MUs, beam delivery time, and gamma passing rates. Statistical analysis was performed using SPSS 17.0.

Results

In plan S_{100}, the $D_{98\%}$ for target PCTV2 decreased by more than 4% in four patients compared to the base plan. In plan S_{80}, the D_{max} for target PGTV and PGTVnd and $D_{98\%}$ for target PCTV2 decreased by more than 3% but less than 4% in two patients, while other target dose parameters changed by less than 2%. Compared to the base plan, plan S_{50}, S_{80}, and S_{100} increased the D_{max} for the brainstem, with statistically significant differences ($P < 0.05$), but the maximum increase did not exceed 1.5%. Plan S_{50} reduced the $D_{50\%}$ and D_{mean} for both parotid glands, while plan S_{80} reduced the D_{mean} for both parotids and the $D_{50\%}$ for the left parotid, with statistically significant differences ($P < 0.001$). Plan S_{100} increased the $D_{50\%}$ and D_{mean} for both parotids and the D_{max} for the spinal cord, with statistically significant differences ($P < 0.05$). Plan S_{80} and S_{100} increased the V_{40} for the thyroid, with statistically significant differences ($P < 0.05$). Compared to the base plan, plan S_{50}, S_{80}, and S_{100} reduced MUs by an average of 5.1%, 21.4%, and 30.9%, respectively, while the average beam delivery time remained around 2.5 minutes for four plans. Under the 1%/1 mm condition, the gamma passing rate for base plan, S_{50}, S_{80}, and S_{100} increased sequentially.

Conclusion

Applying MUs optimization in VMAT plan for NPC can effectively reduce MUs while maintaining target dose coverage and OAR protection, improving gamma passing rate under 1%/1 mm conditions.

Introduction

Nasopharyngeal carcinoma (NPC) is one of the most common malignant cancers in China, particularly in the southeastern regions, where the incidence rate ranges from 20 to 50 per 100,000 people[1]. Due to the complexity of its anatomical structure and sensitivity to radiation, radiotherapy has been the primary treatment method[2]. Radiotherapy techniques have evolved from three-dimensional conformal radiotherapy (3DCRT) to fixed-field intensity-modulated radiotherapy (IMRT), and now to volumetric modulated arc therapy (VMAT)[3]. These advancements have significantly improved the treatment outcomes for NPC[4, 5], with VMAT, in particular, demonstrating the ability to reduce normal tissue exposure, minimize early adverse effects, and improve survival rates compared to IMRT[6]. VMAT provides excellent target volume coverage while protecting organs at risk(OAR), especially neural structures and salivary glands[7, 8].

VMAT is a treatment technique that continuously adjusts the gantry rotation speed, dose rate, and multi-leaf collimator (MLC) movement during beam delivery[9]. Compared to IMRT, VMAT significantly reduces the number of monitor units (MUs), shortens treatment time, and improves treatment efficiency[10, 11]. Reducing MUs also helps lower the risk of secondary cancers induced by radiotherapy[12]. The Eclipse treatment planning system(TPS)offers various optimization tools, such as generalized equivalent uniform dose optimization, structure dose-volume optimization, normal tissue objective optimization, and the monitor unit objective (MUO) tool [13-16]. The MUO tool, which controls MUs in VMAT plan, consists of three parameters: Minimum MU, Maximum MU, and Strength. Minimum MU sets the lower limit for MUs, while Maximum MU sets the upper limit. Strength defines the priority enforced by the MU reduction goal. Previous studies have applied the MUO tool in VMAT plan for hypopharyngeal, prostate, cervical, breast, and non-small cell lung cancers[17-20], showing that appropriate use of the MUO tool can reduce total MU without significantly altering target and OAR doses. However, research on the application of the MUO tool in NPC VMAT plans is limited. NPC presents unique challenges due to its complex structure, high target dose requirements, and proximity to multiple OARs[21]. This study explores the impact of MU optimization on NPC VMAT plan.

Materials and methods

Patient cohort

After receiving approval from the institutional review board at Gaozhou People's Hospital(GYLLYJ-2021043) , a total of 21 patients with NPC treated using the VMAT technique from October 1, 2020 to March 1, 2021 were retrospectively enrolled for this study. Data for

this retrospective analysis were collected and analyzed between October and December 2024. All patient data used in the study were anonymized, and informed consent was waived since the plans in this study were research use only. The cohort included 18 males and 3 females, aged 31–76 years (median age: 45 years; mean age: 51 ± 12 years). Inclusion criteria: pathologically confirmed NPC and consent to undergo radiotherapy. Exclusion criteria: lack of imaging or pathological results, or cachexia.

CT simulation and target delineation

The patient was immobilized with a thermoplastic head-neck mask and head rest, positioned in a supine posture with arms placed at body sides. CT images were acquired using a Siemens large-bore CT simulator (Siemens AG, Forchheim, Germany), with a scan range from the skull vertex to 5 cm below the aortic arch, with a slice thickness of 3 mm. The acquired CT images were sent to the Varian Eclipse TPS v.13.6 (Varian Medical Systems, Palo Alto, CA) for target and OAR delineation. Target volumes included the gross tumor volume (GTV) based on imaging and clinical findings, bilateral neck lymph node(GTVnd), and low risk of subclinical lesions clinical target volumes (CTV1 and CTV2). These four target volumes were uniformly expanded by 3 mm in all directions to form the PGTV, PGTVnd, PCTV1, and PCTV2. The delineation of OARs primarily encompassed the spinal cord, brainstem, optic chiasm, optic nerves, eyes, lenses, pituitary gland, parotid glands, temporal lobes, and temporomandibular joints, etc.

Dose prescription

A simultaneous integrated boost technique was used, with prescription dose of 7000 cGy for PGTV, 6800 cGy for PGTVnd, 6000 cGy for PCTV1, and 5400 cGy for PCTV2, delivered in a total of 32 fractions, with 5 treatments per week. The dose coverage requirement was 100% of the prescription dose cover at least 95% of the target volume. Dose constraints for OAR were set as follows: brainstem ⩽ 5400 cGy, spinal cord ⩽ 4500 cGy, optic chiasm and nerves ⩽ 5400 cGy, lenses ⩽ 1000 cGy, eyes ⩽ 4500 cGy, For the parotid glands, the mean dose to at least one gland was constrained to ⩽ 2600 cGy, with $D_{50\%}$ < 30 Gy . The temporomandibular joint was limited to < 6000 cGy. All target volume definitions and plan evaluations were conducted in accordance with the Radiation Therapy Oncology Group (RTOG) 0615 protocol[22] and the International Commission on Radiation Units and Measurements (ICRU) Report 83[23].

VMAT planning

For each patient, a dual-arc VMAT plan was designed. At the first arc, the gantry rotating counterclockwise from 179° to 181° with a collimator angle of 10°, and the second arc involved the gantry rotating clockwise from 181° to 179° with a collimator angle of 350°. A 6 MV photon beam with a maximum dose rate of 600 MU/min was used. The plan was optimized using a photon optimization algorithm and named "base", which did not utilize the MUO tool. While maintaining all other parameters of the plan "base" such as field geometry, dose-volume objectives, and weights unchanged, the plan was re-optimized solely using the MUO tool. The parameter "Minimum MU" was set to 0, and "Maximum MU" was set to 30% of the total MUs from the plan "base". The "Strength" parameter was set to 50, 80, and 100, respectively, resulting in plans named "S_{50}" ,"S_{80}" and "S_{100}". Thus, each patient had four plans:

"base", "S_{50}," "S_{80}", and "S_{100}".

Comparison of target and oar doses, MUs, and beam delivery time

The re-optimized three plans using the MUO tool (plan S_{50}, S_{80}, and S_{100}) were compared with the base plan in terms of the following parameters: target volume coverage ($D_{95\%}$), maximum dose (D_{max}), mean dose (D_{mean}), and homogeneity index(HI)[23], For OARs, comparisons included the maximum dose (D_{max}) to the brainstem, spinal cord, lenses, eyes, optic chiasm, optic nerves, and pituitary gland, as well as the mean dose to the parotid glands, thyroid gland, and submandibular glands. Additionally, the $D_{50\%}$ for the parotid glands and V_{40} (the volume percentage receiving at least 40 Gy) for the thyroid gland were evaluated. The MUs and total beam delivery time for each plan were also recorded. The beam delivery time was measured using a calibrated stopwatch, focusing solely on the time from beam-on to beam-off for each arc, excluding setup time and gantry rotation time. The total beam delivery time was the sum of the delivery times for both arcs.

Pretreatment plan verification

For pre-treatment plan quality verification, the three-dimensional(3D) dose distributions of the four VMAT plans for each patient were measured using the Sun Nuclear ArcCheck phantom(Sun Nuclear Inc, Melbourne, FL) on a Varian Trilogy linear accelerator(Varian Medical Systems, Palo Alto, CA). The measured 3D dose distributions were compared with those calculated by the TPS using gamma index analysis. Global absolute dose gamma evaluation was performed using SNC Patient software(Sun Nuclear Inc, Melbourne, FL) with a 3%/3 mm criterion and a 10% threshold[24]. Prior to ArcCheck measurements, the output dose of the Trilogy linear accelerator was calibrated according to the International Atomic Energy Agency TRS-398 protocol[25]. Additionally, to investigate whether the gamma passing rate (GPR) improved with reduced MUs, gamma analysis was performed under three different criteria: 2%/2 mm, 3%/2 mm, and 1%/1 mm.

Statistical analysis

Statistical analysis was performed using SPSS 17.0 (IBM, USA) software to compare plan S_{50}, S_{80}, and S_{100} with the base plan. The normality of the data distribution was first assessed. For data conforming to a normal distribution, results were expressed as mean ± standard deviation ($\bar{x} \pm s$). Paired t-tests were conducted to evaluate differences between the plans, with a significance level set at $P < 0.05$, indicating statistically significant differences.

Results

Target dose comparison

The comparison of target dose parameters among the four different plans is presented in Tables 1 and 2. The relative differences in target dose parameters for plan S_{50}, S_{80}, and S_{100} compared to the base plan for 21 patients are illustrated in Figures 1 to 3, respectively.

For plan S_{50}, statistically significant differences ($P < 0.05$) were observed in the D_{mean} of PGTV, $D_{95\%}$ and HI of PGTVnd, and D_{mean} and HI of PCTV2 compared to the base plan. No

significant differences (P > 0.05) were found for other dose parameters.

For plan S_{80}, statistically significant differences (P < 0.05) were noted in the $D_{95\%}$ and HI of PGTV, $D_{95\%}$, D_{mean}, and HI of PGTVnd, and $D_{95\%}$, D_{max}, and HI of PCTV2 compared to the base plan. Other dose parameters showed no significant differences (P > 0.05).

For plan S_{100}, only the D_{max} and D_{mean} of PGTV, D_{max} of PGTVnd, and D_{max} of PCTV1 showed no statistically significant differences (P > 0.05), while other parameters showed significant differences (P < 0.05) compared with the base plan. As shown in Figure 3, the $D_{98\%}$ of PCTV2 in plan S_{100} decreased by more than 4% compared to the base plan in 4 patients. In plan S_{80}, the D_{max} of PGTV and PGTVnd and $D_{98\%}$ of PCTV2 decreased by more than 3% but less than 4% in in 1 out of 21 patients compared to the base plan. For other target dose parameters, the changes were mostly within 2%. The cross-sectional dose distribution and dose volume histograms (DVH) of each structure for one patient are shown in Figure 4 and 5, respectively. As can be seen from Figure 4, the 40 Gy isodose line for plan S_{50}, S_{80}, and S_{100} increasingly extends towards the mandible compared to the base plan.

Table 1. Dose comparisons of target PGTV and PGTVnd for plan s50, s80, s100 vs. base plan（*n*=21）

plan	PGTV				PGTVnd			
	$D_{95\%}$/cGy	D_{max}/cGy	D_{mean}/cGy	HI	$D_{95\%}$/cGy	D_{max}/cGy	D_{mean}/cGy	HI
base	7 080.0± 13.0	7 550.3± 68.8	7 230.4± 11.7	0.047± 0.004	6 892.3± 19.2	7 455.5± 135.7	7 082.7± 32.5	0.061± 0.007
S_{50}	7 074.4± 15.56	7 548.0± 66.2	7 218.3± 8.9	0.046± 0.005	6 882.0± 23.9	7 441.0± 122.4	7 079.0± 35.9	0.063± 0.008
S_{80}	7 069.9± 18.6	7 531.4± 67.7	7 230.8± 14.1	0.050± 0.005	6 857.2± 24.7	7 429.6± 134.6	7 065.8± 34.8	0.068± 0.012
S_{100}	7 050.4± 28.0	7 231.9± 14.5	7 231.9± 14.5	0.055± 0.008	6 822.4± 51.6	7 443.3± 107.3	7 041.4± 44.2	0.075± 0.014
P_1	0.076	0.800	0.002	0.205	0.008	0.285	0.281	0.012
P_2	0.008	0.182	0.912	0.002	<0.001	0.109	<0.001	0.001
P_3	<0.001	0.088	0.683	<0.001	<0.001	0.409	<0.001	<0.001

Note: P_1 indicates the test results of plan S_{50} and base plan; P_2 indicates the test results of plan S_{80} and base plan. P_3 indicates the test result of plan S_{100} and base plan.

Table 2. Dose comparisons of target PCTV1 and PCTV2 for plan s50, s80, s100 vs. base plan（*n*=21）

plan	PCTV1				PCTV2			
	$D_{95\%}$/cGy	D_{max}/cGy	D_{mean}/cGy	HI	$D_{95\%}$/cGy	D_{max}/cGy	D_{mean}/cGy	HI
base	6 261.4± 105.4	7 556.3± 74.0	6 915.8± 83.8	0.174± 0.014	5 513.6± 23.4	7 592.8± 88.5	6 236.2± 69.6	0.311± 0.005
S_{50}	6 269.7± 98.4	7 548.8± 66.6	6 915.5± 80.6	0.172± 0.012	5 520.7± 26.9	7 576.8± 75.2	6 248.3± 71.7	0.307± 0.006
S_{80}	6 249.9± 106.7	7 531.9± 67.3	6 913.9± 82.2	0.176± 0.015	5 479.3± 38.6	7 555.1± 92.9	6 233.4± 72.6	0.318± 0.010
S_{100}	6 214.4± 114.0	7 535.0± 68.4	6 902.9± 83.9	0.184± 0.017	5 417.5± 66.2	7 564.3± 68.2	6 198.0± 81.5	0.334± 0.018
P_1	0.235	0.465	0.882	0.139	0.127	0.170	<0.001	<0.001
P_2	0.145	0.127	0.481	0.060	<0.001	0.033	0.462	0.002
P_3	<0.001	0.081	<0.001	<0.001	<0.001	0.047	<0.001	<0.001

Note: P_1 indicates the test results of plan S_{50} and base plan; P_2 indicates the test results of plan S_{80} and base plan. P_3 indicates the test result of plan S_{100} and base plan.

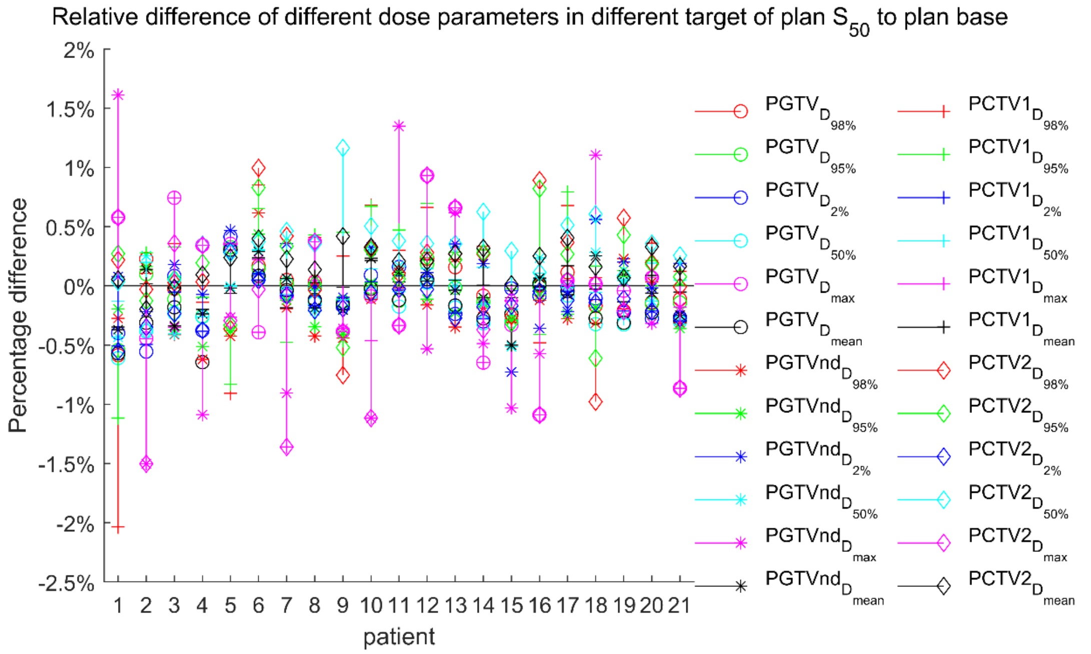

Fig 1. The relative difference between the target dose of plan S_{50} and base plan in 21 patients (n=21)

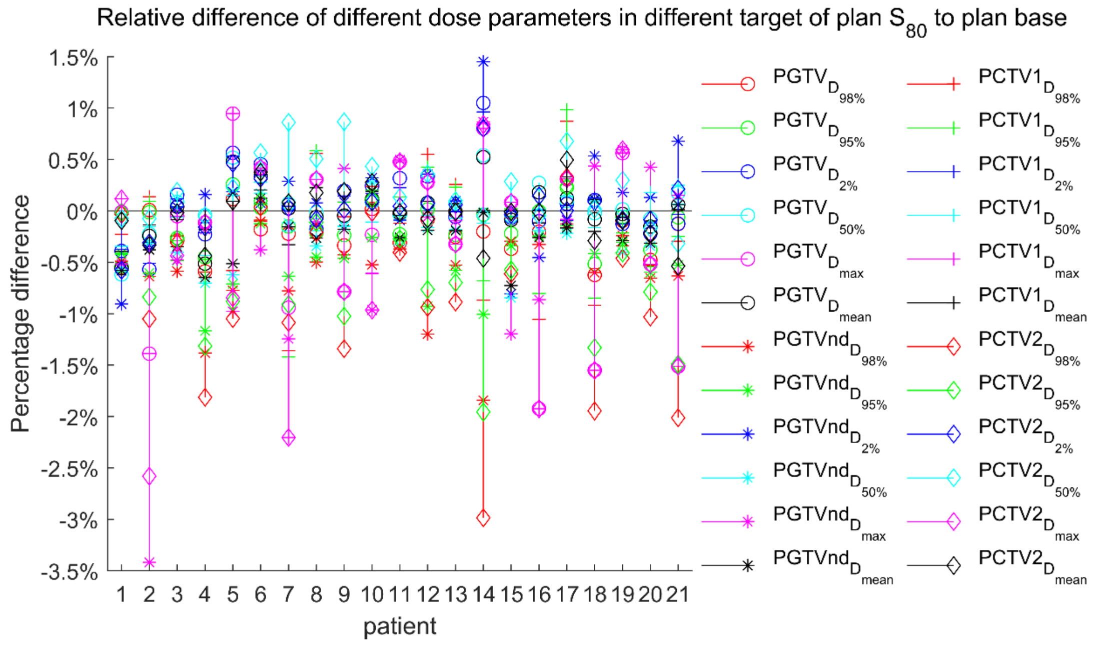

Fig 2. The relative difference between the target dose of plan S_{80} and base plan in 21 patients (n=21)

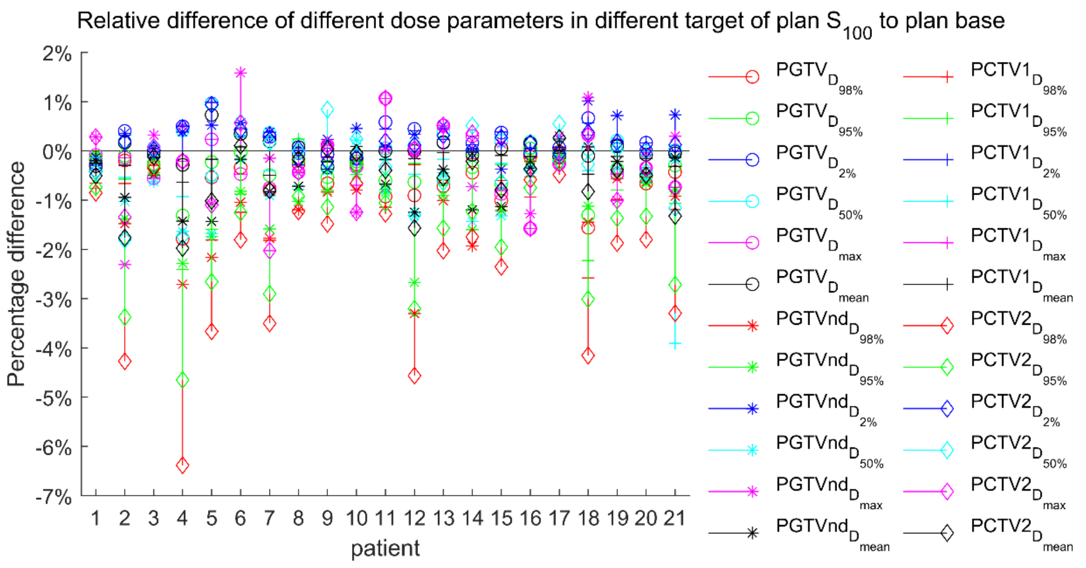

Fig 3. The relative difference between the target dose of plan S_{80} and base plan in 21 patients (n=21)

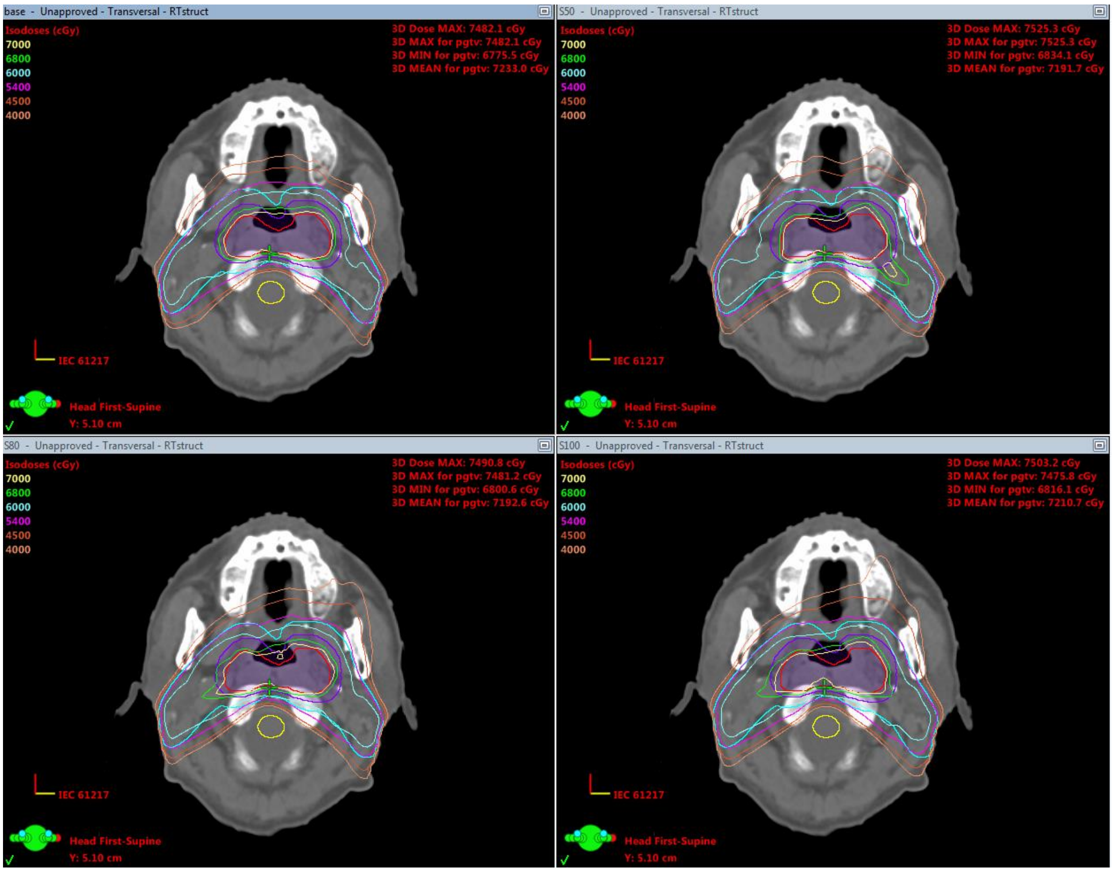

Fig 4. Cross-sectional dose distribution of one patient, with the top left, top right, bottom left, and bottom right indicating planned base, S_{50}, S_{80}, and S_{100}, respectively.

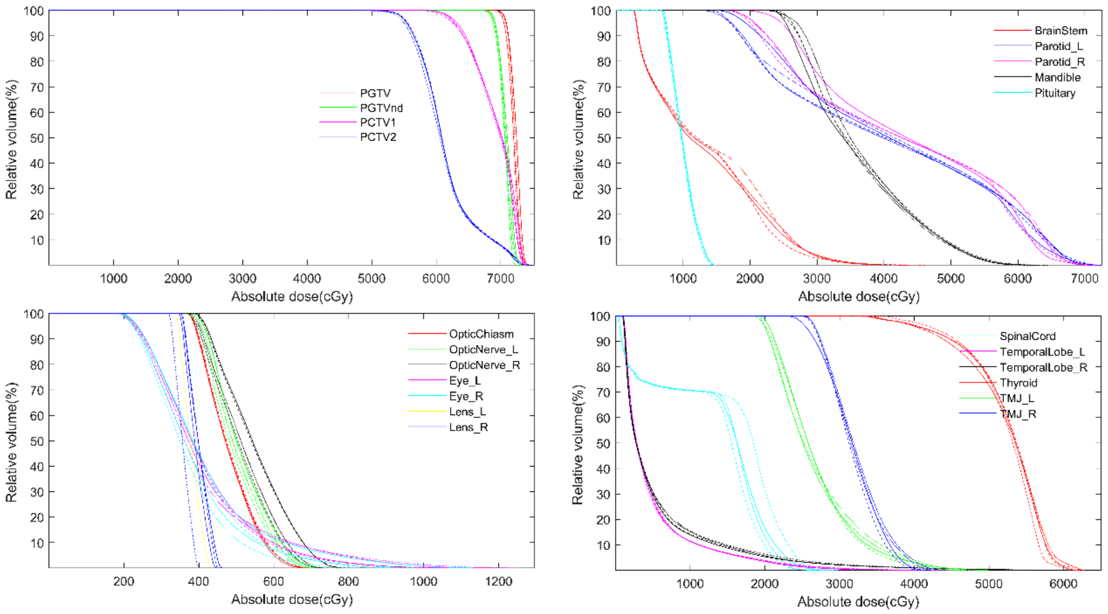

Fig 5. DVH of each structure of four plans for one patient, where "-solid line" represents base plan, "-- long dashed line" represents plan S_{50}, "-.dotted dash line" represents plan S_{80}, and ":dotted line" represents plan S_{100}.

OARs dose comparison

The results of OAR dose comparison between plan S_{50}, S_{80}, S_{100} and base plan are shown in Table 3 and 4. Compared to the base plan, plan S_{50}, S_{80}, and S_{100} increased the D_{max} for the brainstem, with statistically significant differences ($P < 0.05$), but the maximum increase did not exceed 1.5%. Plan S_{50} significantly reduced the $D_{50\%}$ and D_{mean} for both parotid glands ($P < 0.001$). Plan S_{80} significantly reduced the D_{mean} for both parotids and the $D_{50\%}$ for the left parotid ($P < 0.001$), but no significant difference was observed for the $D_{50\%}$ of the right parotid gland ($P = 0.181$). In contrast, plan S_{100} increased the $D_{50\%}$ and D_{mean} for both parotids and the D_{max} for the spinal cord compared to the base plan, with statistically significant differences ($P < 0.05$). Both plan S_{80} and S_{100} increased the V_{40} for the thyroid relative to base plan, which is statistical significant ($P < 0.05$). For the D_{max} of the left and right temporal lobes, the D_{max} of the left and right temporomandibular joints, and D_{mean} of the submandibular gland, no statistically significant differences were observed among plan S_{50}, S_{80}, S_{100}, and the base plan ($P > 0.05$).

Table 3. Dose comparison of brainstem, lens, chiasma, optic nerve, pituitary gland and spinal cord for plan s50, s80, s100 vs. base plan（*n*=21）

plan	brainstem D_{max}/cGy	left lens D_{max}/cGy	right lens D_{max}/cGy	optic chiasma D_{max}/cGy	left optic nerve D_{max}/cGy	right optic nerve D_{max}/cGy	pituitary D_{max}/cGy	spinal cord D_{max}/cGy
base	5 512.1± 648.7	682.5± 187.9	668.9± 156.5	3 807.8± 2 359.4	3 394.8± 2 152.8	4 215.2± 1 820.8	5 093.6± 1 825.0	2 873.4± 299.1
S_{50}	5 566.8± 615.8	681.0± 171.0	676.5± 162.0	3 798.9± 2 398.3	3 331.6± 2 188.9	4 230.5± 1 861.1	5 109.0± 1 834.6	2 755.0± 356.4
S_{80}	5 562.5± 619.1	681.3± 180.7	666.2± 185.0	3 775.9± 2 401.2	3 366.6± 2 217.8	4 228.7± 1 856.0	5 112.6± 1 854.5	2 920.3± 369.9
S_{100}	5 590.1± 631.0	693.8± 227.6	670.2± 213.4	3 787.0± 2 418.7	3 338.4± 2 219.1	4 220.4± 1 860.6	5 128.9± 1 852.4	3 317.2± 433.7

P_1	0.025	0.839	0.231	0.644	0.029	0.455	0.605	0.016
P_2	0.024	0.865	0.850	0.337	0.422	0.392	0.650	0.334
P_3	0.013	0.396	0.944	0.472	0.208	0.820	0.404	<0.001

Note: P_1 indicates the test results of plan S_{50} and base plan; P_2 indicates the test results of plan S_{80} and base plan. P_3 indicates the test result of plan S_{100} and base plan.

Table 4. Dose comparison of parotid gland, temporal lobe, temporomandibular joint, thyroid gland and submandibular gland for plan s50, s80, s100 vs. base plan (n=21)

	left parotid gland		right parotid gland		left temporal lobe	right temporal lobe	left temporomandibular joint	right temporomandibular joint	thyroid gland		submandibular gland
	$D_{50\%}$/cGy	D_{mean}/cGy	$D_{50\%}$/cGy	D_{mean}/cGy	D_{max}/cGy	D_{max}/cGy	D_{max}/cGy	D_{max}/cGy	D_{mean}/cGy	V_{40}/%	D_{mean}/cGy
base	3 228.0±443.8	3 649.4±310.6	3 551.8±618.2	3 873.4±426.7	6 835.4±820.8	6 970.2±373.9	5 235.8±939.2	4 970.6±685.4	4 955.2±225.1	87.2±7.9	5 779.7±384.4
S_{50}	3 094.4±520.0	3 549.9±321.7	3 439.1±728.7	3 762.9±451.8	6 880.3±821.9	7 003.0±354.2	5 204.5±962.7	4 963.0±769.5	5 017.6±335.5	88.0±7.4	5 800.7±391.4
S_{80}	3 145.3±480.2	3 591.3±310.5	3 505.5±686.4	3 811.2±454.0	6 859.8±847.6	6 983.0±337.3	5 237.4±899.6	4 972.2±750.0	4 787.4±1 005.0	90.6±6.5	5 751.4±390.9
S_{100}	3 431.3±440.8	3 767.4±310.4	3 684.8±624.4	3 962.7±425.4	6 808.1±841.4	6 949.9±367.6	5 224.1±817.2	4 992.9±731.8	5 108.3±287.0	94.5±5.0	5 737.8±422.9
P_1	<0.001	<0.001	0.004	<0.001	0.134	0.273	0.494	0.908	0.286	0.312	0.066
P_2	0.001	<0.001	0.181	<0.001	0.117	0.602	0.971	0.973	0.414	0.002	0.059
P_3	<0.001	<0.001	0.003	0.005	0.058	0.467	0.810	0.706	0.006	<0.001	0.062

Note: P_1 indicates the test results of plan S_{50} and base plan; P_2 indicates the test results of plan S_{80} and base plan. P_3 indicates the test result of plan S_{100} and base plan.

MU and beam delivery time comparison

The MUs and beam on time of the four plans are shown in Table 5. The plan S_{50}, S_{80}, and S_{100}, which utilized the MUO tool, showed a statistically significant reduction in MUs compared to the base plan ($P<0.05$), with average decreases of 5.1%, 21.5%, and 30.9%, respectively. However, it was observed that despite the reduction in MUs, there was no

significant decrease in the beam-on time for the plans. The average beam-on time for all four plans remained around 2.5 minutes.

Table 5. MUs and beam on time for plan s50, s80, s100 vs. base plan (n=21)

	MUs	Time(min)
base	670±84.7	2.50±0.05
S_{50}	636±57.9	2.50±0.06
S_{80}	526±61.9	2.48±0.05
S_{100}	463±65.0	2.48±0.05
t_1	3.239	0.977
P_1	0.004	0.340
t_2	9.652	-0.568
P_2	<0.001	0.576
t_3	10.403	-0.204
P_3	<0.001	0.841

Note: P_1 indicates the test results of plan S_{50} and base plan; P_2 indicates the test results of plan S_{80} and base plan. P_3 indicates the test result of plan S_{100} and base plan.

Gamma passing rate comparison

The GPR of the four plans under different evaluation conditions are shown in Table 6. There was no significant difference in GPR between plan S_{50} and base plan under the four evaluation conditions (P >0.05). The difference between plan S_{80} and plan S_{100} relative to base plan was statistically significant in the four evaluation conditions (P<0.001). Combining the data from Table 5, where the MUs for base plan, S_{50}, S_{80}, and S_{100} decreased sequentially, and from Table 6, under the 1%/1 mm criterion, it was observed that the reduction in MUs was associated with an improvement in the GPR of the plans.

Table 6. Gamma pass rates of the four plans under different evaluation conditions

plan	3% /3 mm	2%/2 mm	3% /2 mm	1% /1 mm
base	99.7%±0. 3%	98.3%±1. 0%	99.4%±0. 5%	86.7%±4. 2%
S_{50}	99.6%±0. 3%	98.4%±1. 2%	99.4±0. 4%	87.1%±4. 4%
S_{80}	99.8%±0. 2%	99.2%±0. 8%	99.7%±0. 3%	90.4%±4. 7%
S_{100}	99.8%±0. 2%	99.3%±1. 0%	99.7%±0. 4%	92.3%±5. 1%
t_1	0.188	-0.401	0.167	-0.653
P_1	0.853	0.693	0.869	0.522
t_2	-3.414	-6.598	-4.077	-6.327
P_2	0.003	<0.001	0.001	<0.001
t_3	-3.111	-5.678	-3.313	-7.191
P_3	0.006	<0.001	0.004	<0.001

Note: P_1 indicates the test results of plan S_{50} and base plan; P_2 indicates the test results of plan S_{80} and base plan. P_3 indicates the test result of plan S_{100} and base plan.

Discussion

Radiotherapy plays a crucial role in the treatment of NPC, with IMRT showing significant long-

term efficacy and a 5-year local control rate of approximately 90%[26]. Particularly, the clinical application of VMAT has demonstrated advantages over IMRT, not only providing superior dose distribution to the target but also effectively protecting the OARs[27]. The utilization of VMAT in radiotherapy for NPC can reduce treatment-related late adverse effects such as ototoxicity, trismus, and temporal lobe injury [28-30].

Varian provided the function and definition of the MUO tool without further elaboration on its application methods. Jiménez-Puertas et al. conducted a retrospective analysis of 40 prostate, 20 gynecological, and 20 head and neck VMAT plans by altering the Maximum MU parameter value of the MUO tool. They discovered that for all three studied sites, the plans exhibited the greatest reduction in MUs when the Maximum MU was set to 400, without significant changes to the original dose distribution[15]. However, their study did not investigate the impact of altering the Strength parameter value on plan outcomes. Clemente et al. selected 7 prostate VMAT plans, setting the Strength to 50 and 100, the Minimum MU to 0% and 50% of the baseline plan's MUs, and the Maximum MU to 25%, 50%, and 75% of the baseline plan's MUs. Compared to the baseline plans, they found that using the MUO tool could reduce MUs by an average of 28%[18]. Huang et al. applied the MUO tool to non-small cell lung cancer stereotactic body radiation therapy plans, demonstrating that the tool could maintain or improve OAR doses while significantly reducing plan MUs[19]. However, these studies have certain limitations, such as a small number of cases, not altering the Strength parameter value of the MUO tool, and not studying the GPR of plans after MUs reduction. This study utilizes the MUO tool to optimize nasopharyngeal carcinoma VMAT plans and evaluates the impact on dose distribution, MUs, beam-on time, and pre-treatment plan quality verification results. While plans meeting clinical requirements can be obtained without using the MUO tool during plan optimization, its use can significantly reduce plan MUs while maintaining target dose coverage and OAR protection. This study found that plans optimized with the MUO tool (S_{50}, S_{80}, and S_{100}) significantly reduced MUs compared to the base plan without MUO optimization (with P=0.004, P<0.001, P<0.001, respectively). The plan S_{100} showed the greatest reduction, reaching 30.9%. However, for the plan S_{100}, 8 out of 21 patients had a $D_{95\%}$ for the target PCTV2 less than 5400 cGy, not meeting clinical requirements. Some individual target doses in the plan S_{50} and S_{80} showed statistically significant differences compared to the base plan (P<0.05), but these were not clinically significant, as deviations from the base plan were mostly less than 1%, this indicates an absolute dose deviation of under 50 cGy , which means 1-2 cGy per fraction over 33 fractions, thus having limited clinical impact. The use of the MUO tool significantly reduced the target's HI, possibly due to reduced plan modifiability, sacrificing target uniformity to ensure dose coverage. The reduction in MUs might lead to an increase in the D_{max} for some OARs like the brainstem and spinal cord, but the maximum increase did not exceed 1.5%. This could be because the reduction in MUs increases the value per cGy/MU, and to maintain the dose to the brainstem and spinal cord without significant changes, slightly higher weights could be assigned to these structures during re-optimization with the MUO tool compared to the base plan.

The reduction in MUs did not result in a significant change in the beam-on time of the plans, with the beam-on time for all four plans around 2.5 minutes. This is because the photon optimization algorithm of the Eclipse TPS tends to maintain the gantry rotation speed of VMAT plan at approximately 6.0°/s. With the rotation speed remaining largely unchanged,

the adjustment of VMAT plan is achieved by altering the dose rate and leaf motion speed. Generally, the quality of a treatment plan is directly related to the plan's MUs. Three-dimensional dose measurements conducted to evaluate the dose distribution showed that as the plan's MUs decreased, there was an improvement in the GPR before treatment. In this study, under the evaluation criteria of 1%/1 mm, the GPR for plan S_{50}, S_{80}, and S_{100} improved by an average of 0.4%, 3.7%, and 5.6%, respectively, compared to the base plan. This may be due to the generation of simpler MLC shapes and dose rate conversion patterns, and also less out-of-field dose. This implies that for complex plans (such as whole pelvic irradiation) with a GPR below 95% under 3 mm/3% conditions, the MUO tool can be used to reduce their MUs, thereby improving plan quality. The scatter dose received by patients is directly related to the number of MUs delivered by the accelerator during treatment, and fewer MUs mean reduced out-of-field scatter dose from the linear accelerator, thus lowering the risk of secondary radiation to patients [31].

This study has several limitations. Firstly, it is a retrospective study that only explores the impact of using the MUO tool for optimization in nasopharyngeal carcinoma VMAT plan on the dose distribution of target areas and OARs, beam-on time, and GPR. It does not investigate the effects on tumor control probability and normal tissue complication probability. Secondly, the advantages of using the MUO tool need to be confirmed through long-term patient follow-up and based on a large number of case practices.

Conclusion

In the VMAT planning for nasopharyngeal carcinoma, the rational use of the MUO tool can reduce the MUs while maintaining plan quality comparable to the base plan. Among the three plans S_{50}, S_{80}, and S_{100} generated using the MUO tool in this study, the plan S_{50} was able to reduce the MUs without significant changes in the dose distribution to the target and OARs. However, the S_{80} and S_{100} plan may result in underdosing in certain target.

References

1. Imano N, Murakami Y, Nakashima T, Takeuchi Y, Takahashi I, Doi Y, et al. Clinical outcomes of concurrent chemoradiotherapy with volumetric modulated arc therapy in patients with locally advanced nasopharyngeal carcinoma. Jpn J Radiol. 2017;35(11):673-80. Epub 2017/09/10. doi: 10.1007/s11604-017-0680-5. PubMed PMID: 28887732.

2. U A, C K, ND K, Ozkaya K, H B, M A. Nasopharyngeal carcinoma radiotherapy with hybrid technique. Med Dosim. 2019;44(3):251-7. doi: 10.1016/j.meddos.2018.09.003.

3. Mohan G, T P AH, A J J, K M SD, Narayanasamy A, Vellingiri B. Recent advances in radiotherapy and its associated side effects in cancer—a review. The Journal of Basic and Applied Zoology. 2019;80(1). doi: 10.1186/s41936-019-0083-5.

4. YH L, YY S, KM L, LC W, IWK T, FCH H. Long-term outcomes after reirradiation in nasopharyngeal carcinoma with intensity-modulated radiotherapy: A meta-analysis. . . Head Neck. 2018;40(3):622-31. doi: 10.1002/hed.24993.

5. XS S, XY L, QY C, LQ T, HQ M. Future of Radiotherapy in Nasopharyngeal Carcinoma. Br J

Radio. 2019;92(1102):20190209.
6. Guo R, Tang LL, Mao YP, Zhou GQ QZ, Liu LZ, Lin AH, Liu MZ, Ma J, Sun Y. . Clinical Outcomes of Volume-Modulated Arc Therapy in 205 Patients with Nasopharyngeal Carcinoma: An Analysis of Survival and Treatment Toxicities. . PLoS One 2015 J;10(7). doi: 10.1371/journal.pone.0129679.
7. Amaoui B, Semghouli S, Mouhssine D, Bayla A, Bouih, A., Bouih, N., Tamri, H., Mourchid, M. E., & Benjaafar, N. . Dosimetric analysis of VMAT Irradiation in Nasopharyngeal Carcinoma: A Moroccan Radio Oncology Center's experience. . European Journal of Molecular & Clinical Medicine. 2021;8(3):979-88.
8. Franzese C, Fogliata A, Loi M, Badalamenti M FD, Comito T, Cozzi L, Reggiori G, Tomatis S, Scorsetti M. Dosimetric impact of volumetric modulated arc therapy for nasopharyngeal cancer treatment. Reports of practical oncology and radiotherapy : journal of Greatpoland Cancer Center in Poznan and Polish Society of Radiation Oncology. 2021 26(1):101-10. doi: 10.5603/rpOr.a2021.0017.
9. K. O. Volumetric modulated arc therapy: IMRT in a single gantry arc Medical physics. 2008;35(1):310-7. doi: 10.1118/1.2818738. PubMed Central PMCID: PMC18293586.
10. Wang Q, Qin J, Cao R, Xu T YJ, Zhu S, Wu J, Xu G, Zhu L, Jiang W, Li W, Xiong W. . Comparison of Dosimetric Benefits of Three Precise Radiotherapy Techniques in Nasopharyngeal Carcinoma Patients Using a Priority-Classified Plan Optimization Model. Front Oncol 2021 Sep 27;11:646584. doi: 10.3389/fonc.2021.646584.
11. Amin, Naima & Atiq, Atia & Lali, Muhammad Ikram & Atiq MN, Hamza & Farooq, Atiqa & Yousaf, Muhammad & Iqbal, Khalid & Akbar, Anzal. Dosimetric analysis of Rapid Arc (VMAT) treatment planning in head and neck cancer for quality assurance treatment. Journal of King Saud University - Science 2022;35. 102476. . doi: 10.1016/j.jksus.2022.102476.
12. Ho HW, Lee SP, Lin HM, al. e. Dosimetric comparison between RapidArc and HyperArc techniques in salvage stereotactic body radiation therapy for recurrent nasopharyngeal carcinoma. Radiat Oncol. 2020;15(1):164
doi: 10.1186/s13014-020-01602-7.
13. Fogliata A, Thompson S, Stravato A, Tomatis S, Scorsetti M, Cozzi L. On the gEUD biological optimization objective for organs at risk in Photon Optimizer of Eclipse treatment planning system. Journal of applied clinical medical physics. 2017;19(1):106-14. doi: 10.1002/acm2.12224.
14. Bell JP, Patel P, Higgins K, McDonald MW, Roper J. Fine-tuning the normal tissue objective in eclipse for lung stereotactic body radiation therapy. Medical Dosimetry. 2018;43(4):344-50. doi: 10.1016/j.meddos.2017.11.004.
15. Jimenez-Puertas S, Sanchez-Artunedo D, Hermida-Lopez M. Assessment of the Monitor Unit Objective tool for VMAT in the Eclipse treatment planning system. Reports of practical oncology and radiotherapy : journal of Greatpoland Cancer Center in Poznan and Polish Society of Radiation Oncology. 2018;23(2):121-5. Epub 2018/03/21. doi: 10.1016/j.rpor.2018.02.001. PubMed PMID: 29556140; PubMed Central PMCID: PMCPMC5856680.
16. M O, I G, C P, W A, WA B. Analysis of RapidArc optimization strategies using objective function values and dose-volume histograms. Journal of applied clinical medical physics. 2009;11(1):3114. doi: 10.1120/jacmp.v11i1.3114.
17. Ahamed S, Singh N, Gudipudi D, Mulinti S, Talluri A, Soubhagya B, et al. Assessment of monitor unit limiting strategy using volumetric modulated arc therapy for cancer of hypopharynx. Phys Med. 2017;35:73-80. Epub 2017/02/24. doi: 10.1016/j.ejmp.2017.01.016. PubMed PMID:

28228330.
18. Clemente S, Cozzolino M, Chiumento C, Fiorentino A CR, Fusco V. . Monitor unit optimization in RapidArc plans for prostate cancer. . Journal of applied clinical medical physics. 2013;14(3):4114. doi: 10.1120/jacmp.v14i3.4114.
19. Huang BT, Lin Z, Lin PX, Lu JY CC. Monitor unit optimization in stereotactic body radiotherapy for small peripheral non-small cell lung cancer patients. . Sci Rep. 2015;5:18453. doi: 10.1038/srep18453.
20. Mancosu P, Reggiori G, Alongi F, Cozzi L, Fogliata A, Lobefalo F, et al. Total monitor units influence on plan quality parameters in volumetric modulated arc therapy for breast case. Phys Med. 2014;30(3):296-300. Epub 2013/10/01. doi: 10.1016/j.ejmp.2013.08.142. PubMed PMID: 24076257.
21. Zhang A, Gao L, Shi X, Kang. W. Research Progress of Radiotherapy for Nasopharyngeal Carcinoma. International Journal of Frontiers in Medicine. 2022;4(8):10-2. doi: 10.25236/ijfm.2022.040803.
22. Lee NY, Zhang Q, Pfister DG, Kim J, Garden AS, Mechalakos J, et al. Addition of bevacizumab to standard chemoradiation for locoregionally advanced nasopharyngeal carcinoma (RTOG 0615): a phase 2 multi-institutional trial. The Lancet Oncology. 2012;13(2):172-80. doi: 10.1016/s1470-2045(11)70303-5.
23. 83: HNDI-R. Verordnung, Dokumentation und Kommunikation der fluenzmodulierten Photonenstrahlentherapie (IMRT) [The ICRU Report 83: prescribing, recording and reporting photon-beam intensity-modulated radiation therapy (IMRT)]. Strahlentherapie und Onkologie : Organ der Deutschen Rontgengesellschaft [et al]. 2012;88(1):97-9.
24. Low DA, Harms WB, Mutic S, JA. P. A technique for the quantitative evaluation of dose distributions. . Medical physics. 1998;25(5):656-61.
25. INTERNATIONAL ATOMIC ENERGY AGENCY, Absorbed Dose Determination in External Beam Radiotherapy, Technical Reports Series No. 398 (Rev. 1), IAEA, Vienna (2024), . doi: https://doi.org/10.61092/iaea.ve7q-y94k.
26. Gou X, Duan B, Shi H, Qin L XJ, Chen N. . The relations of dosimetric parameters with long-term outcomes and late toxicities in advanced T-stage nasopharyngeal carcinoma with IMRT. . Head Neck. 2020;42(1):85-92. doi: 10.1002/hed.25986.
27. Lu S, Fan H, Hu X ea. Dosimetric Comparison of Helical Tomotherapy, Volume-Modulated Arc Therapy, and Fixed-Field Intensity-Modulated Radiation Therapy in Locally Advanced Nasopharyngeal Carcinoma. Frontiers in oncology. 2021;11:764946. doi: 10.3389/fonc.2021.764946.
28. He L XJ, Wei Z, He Y, Wang J, Guan H, Mu X, Peng X. Toxicity and dosimetric analysis of nasopharyngeal carcinoma patients undergoing radiotherapy with IMRT or VMAT: A regional center's experience. . Oral Oncol 2020 109:104978.
29. Lee HF, Lan JH, Chao PJ, al. e. Radiation-induced secondary malignancies for nasopharyngeal carcinoma: a pilot study of patients treated via IMRT or VMAT. Cancer Manag Res. 2018;10:131-41. doi: 10.2147/CMAR.S145713.
30. Huang TL, Tsai MH, Chuang HC, al. e. Quality of life and survival outcome for patients with nasopharyngeal carcinoma treated by volumetric-modulated arc therapy versus intensity-modulated radiotherapy. Radiat Oncol. 2020;15(1):84. doi: 10.1186/s13014-020-01532-4.
31. Patel S, Drodge S, Jacques A, Warkentin H, Powell K, Chafe S. A Comparative Planning Analysis

and Integral Dose of Volumetric Modulated Arc Therapy, Helical Tomotherapy, and Three-dimensional Conformal Craniospinal Irradiation for Pediatric Medulloblastoma. Journal of medical imaging and radiation sciences. 2015;46(2):134-40. doi: 10.1016/j.jmir.2014.11.003.
