# Supplementary material for: Impact of monitor unit optimization in volumetric modulated arc therapy planning for nasopharyngeal carcinoma
Source: PLoS One. 2025 Jun 25;20(6):e0327153. doi: 10.1371/journal.pone.0327153 (PMC12193012; doi:10.1371/journal.pone.0327153)
Supplement: Attachment — Submitted filename: Response to Reviewers.docx [file pone.0327153.s005.docx]

Dear Editors and Reviewers:

Thank you for your letter and for the reviewers’ comments concerning our manuscript entitled “Research on the Impact of Monitor Unit Optimization in Volumetric Modulated Arc Therapy Planning for Nasopharyngeal Carcinoma” (ID: PONE-D-25-12726).Those comments are all valuable and very helpful for revising and improving our paper, as well as the important guiding significance to our researches. We have studied comments carefully and have made correction which we hope meet with approval.

Revised portion are marked in red in the paper. The main corrections in the paper and the responds to the reviewer’s comments are as fllowing:

Responds to the reviewer’s comments:

**Reviewer #1:**

1. ***-Language and Style: While the manuscript is generally intelligible, some grammatical issues, tense inconsistencies, and awkward phrasing could be improved for clarity and flow. A light language edit by a native English speaker or language service would help.***

This document has undergone comprehensive grammatical refinement and tense optimization, with final polishing conducted by professional English language editors.

2. ***-Discussion Depth: The discussion could benefit from a slightly more in-depth comparison with existing studies that evaluated Strength variation in MUO parameters—notably the clinical implications of underdosing in S100 could be expanded.***

In the Discussion section, we have added comparative analyses with existing studies and further elaborated on the clinical implications of underdosage in Plan S_100_.

3. ***-Figures and Tables: The legends for Figures 1–5 could be made more detailed. Also, a summary figure or schematic explaining the MUO tool settings and their clinical impact would enhance readability.***

The captions of Figures 1 to 5 have been supplemented with additional details. Additionally, a schematic diagram of the MUO tool's parameter settings has been added.

**Reviewer #2:**

***1. In the manuscript draft section, the Author mentioned that the patient's data used in the***

***study was treated between October 23 to March 24. In contrast, the main abstract,***

***manuscript, and ethics statement were mentioned as October 2020 to March 2021.***

The patient data used in this study were collected from those treated between October 2020 and March 2021, not October 2023 to March 2024. The manuscript draft has been revised accordingly and resubmitted.

***2. In the main manuscript, under the heading 'VMAT planning,' how did the author decide to take the maximum MU 30 %?, Can it be more elaborate?***

The maximum MU constraint was set at 30% of the base plan's total MU based on prior studies, which has now been referenced in the manuscript.

3. ***Oar is written in small letters. It must be in capital letters.***

As requested, "Oar" has been corrected to "OAR" in all sections.

***4.*** ***Under the heading “Comparison of the target and OAR doses….” The sentence***

***“excluding setup time and gantry rotation time….” Can be written as excluding the setup time and gantry repositioning time, or beam mode up time.***

As requested, the “gantry rotation time” has been written as “beam mode up time”.

***5. Under the heading “ pretreatment plan verification,” 10% threshold in the 3% 3mm***

***criteria? I doubt it is given in the cited reference 24.***

The literature citations have now been corrected and properly referenced throughout the manuscript.

***6. Table 4 can be rearranged.***

Table 4 has been rearranged to improve clarity and logical flow of the presented data.

***7. In Tables 5 and 6, what are t1, t2, and t3? The author must mention it and elaborate on these terms.***

The t_1_, t_2_, and t_3_ values presented in Tables 5 and 6 have been clearly defined in the corresponding figure captions to ensure proper interpretation.

**Reviewer #3:**

1. ***Clarify whether MUO was applied during initial optimization or as a post-hoc re-optimization. This affects reproducibility and clinical implementation.***

The MUO was implemented during the initial optimization phase before subsequent refinements.

***2. Specify if OAR constraints were adjusted during MUO re-optimization. The observed increases in brainstem/spinal cord Dmax (≤1.5%) suggest constraints may need tightening.***

During MUO re-optimization, the original constraints for both target and OARs remained unchanged, with adjustments applied solely to monitor unit optimization.

***3. Justify the choice of gamma criteria (1%/1 mm, 2%/2 mm, 3%/3 mm). While 1%/1 mm is stringent, its clinical relevance should be discussed (e.g., is 3%/2 mm more practical for routine QA?).***

The clinical significance of applying 1%/1mm gamma criteria was further elaborated, highlighting its stringent sensitivity in detecting teep dose gradients near critical structures compared to more lenient criteria.

***4. For OARs, discuss if the increases in brainstem Dmax (≤1.5%) or thyroid V40 are acceptable given clinical tolerances.***

While the re-optimized plans demonstrated modest increases in brainstem D_max_ and thyroid V_40_, all values remained well within institutional clinical tolerance limits. It has been supplemented in the discussion.

***5. Highlight that S100’ MU reduction (30.9%) comes at the cost of target underdosing in 8/21 patients, making S50 or S80 more viable.***

The discussion has been revised to emphasize that compared to S100, S50 or S80 may represent more feasible compromise solutions***.***

***6. Explain why MU reduction did not shorten delivery time (e.g., fixed gantry speed, dose rate limitations).***

Additional discussion has been incorporated to address why MU reduction did not shorten delivery time.

***7. Fixed MUO parameters (e.g., Maximum MU = 30% of base plan) may not be optimal for all cases. It is suggested to increase the discussion on the influence of the Maximum MU parameter on the results.***

The impact of the Maximum MU parameter on the planning results has been supplemented in the discussion.

***8.Suggest prospective validation with larger cohorts, long-term toxicity outcomes, and exploration of adaptive MUO strategies.***

Future studies will include prospective validation with more cases and long-term toxicity outcome assessments.

***9.In the Abstract, emphasize the improvement in gamma passing rates with MU reduction, as this is a notable practical outcome.***

In the abstract, key emphasis has been placed: the significant improvement in Gamma passing rate achieved by reducing MU (Monitor Units), a result with important clinical significance.

***10.Ensure consistent formatting (e.g., italicize journal names, use "et al." correctly). The citation formats are inconsistent. Some authors use their full names while others use abbreviations.***

The manuscript formatting has been carefully reviewed, and the reference style has been corrected accordingly.
